# Supplementary material for: Content-rich biological network constructed by mining PubMed abstracts
Source: BMC Bioinformatics. 2004 Oct 8;5:147. doi: 10.1186/1471-2105-5-147 (PMC528731; doi:10.1186/1471-2105-5-147)
Supplement: Additional File 2 — The original results of the above study (non-essential files are deleted to keep the file size under the limit set by BMC bioinformatics). [file 1471-2105-5-147-S2.bz2 › chilibotAdditionalFile2/dip30/26ID8999548E89/html/IFNG_IL18.html]

 


 **IFNG** and **IL18** 
  
Found 772 abstracts in PubMed, retrieved 30.  
 

 What does Google say? 
 PDF only 
| .edu only 

---

**Interactive relationship** (e.g. stimulation, inhibition, etc)

**Stimulatory relationship**- Nai ; ve CD4 T cells differentiate into interferon gamma  [ **IFNG** ]  IFN gamma  [ **IFNG** ]  producing Th1 effector cells when stimulated by interleukin 18  [ **IL18** ]  IL 18  [ **IL18** ]  and IL 12.  Ref: 12829194 Cardiovasc Res< MedlineTA>Cardiovasc Res,
- Naive CD4 CD62L Tcells from normal C57BL 6 mice or from DO11.10 Rag2 OVA specific TCR transgenic mice secreted substantial amounts of IFN gamma  [ **IFNG** ]  when stimulated concurrently with high dose IL 2 plus IL 12 or IL 18  [ **IL18** ] .  Ref: 12811838 Eur J Immunol, 2003
- However, because interleukin IL 18  [ **IL18** ]  acts synergistically with IL 12 to augment Th1 responses and interferon IFN gamma  [ **IFNG** ] , we designed an EGT protocol using IL 12 IL 18  [ **IL18** ]  with the aim of enhancing the antitumor effects obtained with IL 12 alone.  Ref: 12820368 Anticancer Res< MedlineTA>Anticancer Res, 2003
- Our data show that enhanced IFN gamma  [ **IFNG** ]  production in the lungs of bleomycin treated mice is at least partly IL 12 and IL 18  [ **IL18** ]  dependent.  Ref: 12857673 Am J Physiol Lung Cell Mol PhysiolAm J Physiol Lung Cell Mol Physiol, 2003
- IL 18  [ **IL18** ]  is a cytokine structurally and functionally related to IL 1 that, in synergy with IL 12, stimulates the synthesis of IFN gamma  [ **IFNG** ]  from T lymphocytes and natural killer cells.  Ref: 12884303 Eur J ImmunolEur J Immunol, 2003
**Neutral relationship**- Interferon IFN gamma  [ **IFNG** ]  inducing factor was previously termed interleukin IL 18  [ **IL18** ] .  Ref: 12792854 J Infect Dis, 2003
- IL 12 and IL 18  [ **IL18** ]  activate the synthesis of IFN gamma  [ **IFNG** ]  by innate immune cells, including NK, NKT and gammadeltaT cells and promote the differentiation of Th1 type acquired immune responses.  Ref: 12825993 Rev Iberoam Micol, 2002
- In the gut of non infected piglets, IL 1 beta, IL 8 and IL 18  [ **IL18** ]  were detected whereas TNF alpha and IFN gamma  [ **IFNG** ]  were mostly missing.  Ref: 12879755 Folia Microbiol (Praha), 2003
- CD4 and CD8 T cells induced IL 18  [ **IL18** ]  from DC and primed for IL 12 p70 secretion via interferon gamma  [ **IFNG** ]  IFN gamma  [ **IFNG** ]  or tumour necrosis factor alpha TNF alpha.  Ref: 12871213 Immunology, 2003
- Interleukin 18  [ **IL18** ]  IL 18  [ **IL18** ]  can act together with IL 12 in promoting the generation of IFN gamma  [ **IFNG** ]  producing Th1 cells.  Ref: 12796406 Clin Cancer Res, 2003
- Interleukin 18  [ **IL18** ]  IL 18  [ **IL18** ] , a monokine that shares functional abilities with IL 12, is a potent inductor of interferon gamma  [ **IFNG** ]  IFN gamma  [ **IFNG** ]  by Th1 and natural killer NK cells.  Ref: 12858406 J Med Virol, 2003
- Moreover, IFN gamma  [ **IFNG** ]  mRNA steady state levels were correlated positively with those of IFNAR 1, IRF 1, and IL 18  [ **IL18** ] , suggesting a coordinated induction of these genes.  Ref: 12794720 J Med Virol< MedlineTA>J Med Virol, 2003
- The protective effect of IL 18  [ **IL18** ]  bp Fc was accompanied by modified ex vivo immune responses, in that spleen cells and peritoneal macrophages contained fewer IFN gamma  [ **IFNG** ]  secreting cells and released lower amounts of nitrite an index of nitric oxide production and IL 1beta.  Ref: 12884303 Eur J ImmunolEur J Immunol, 2003
- Moreover, they deliver additional support for the view that IFN gamma  [ **IFNG** ]  still has to be considered as a mediator that determines the outcome of inflammation, e.g., via its ability to activate IL 18  [ **IL18** ]  expressing cells and to initiate a delayed type hypersensitivity reaction.  Ref: 12794718 J Med Virol, 2003
- These results indicate that combined administration of IL 18  [ **IL18** ]  and low dose IL 2 could be a new model for cancer immunotherapy, which probably engages the activation of natural killer cells through interferon gamma  [ **IFNG** ]  and Fas L dependent pathways.  Ref: 12806277 J Immunother< MedlineTA>J Immunother, 2003
- Anti IL 12 and anti IL 18  [ **IL18** ]  antibodies decreased IFN gamma  [ **IFNG** ]  production by these cells.  Ref: 12857673 Am J Physiol Lung Cell Mol PhysiolAm J Physiol Lung Cell Mol Physiol, 2003
- Thus, blockade of IL 18  [ **IL18** ]  appears to be a viable clinical target to combat the pathologic consequences of sepsis via IFN gamma  [ **IFNG** ]  mechanisms.  Ref: 12792854 J Infect Dis, 2003
- RESULTS IL 12 IL 18  [ **IL18** ]  EGT inhibited tumor growth significantly better than IL 12 EGT, which was consistent with significantly higher intratumoral levels of IFN gamma  [ **IFNG** ] .  Ref: 12820368 Anticancer Res< MedlineTA>Anticancer Res, 2003

**Non-interactive relationship** (e.g. studied together, co-existance, homology, etc.)

- However, mRNA expression for IL 18  [ **IL18** ]  and interferon gamma  [ **IFNG** ]  IFN gamma  [ **IFNG** ]  was downregulated, and the expression of mRNA for the anti inflammatory cytokine transforming growth factor beta4 TGF beta 4 was upregulated.  Ref: 12859858 J Interferon Cytokine Res, 2003
- Expression of the chemokine IP 10 correlates with the accumulation of hepatic IFN gamma  [ **IFNG** ]  and IL 18  [ **IL18** ]  mRNA in chronic hepatitis C but not in hepatitis B.  Ref: 12794718 J Med Virol, 2003
- To investigate the intra hepatic activation of the IFN system in patients affected by chronic HCV infection in comparison with that observed in a non infectious liver disease such as non alcoholic steatohepatitis, we measured the liver steady state mRNA levels of interferon alpha, interferon beta and interferon gamma  [ **IFNG** ]  as well as of IFN related genes IFNAR 1, STAT1alpha, PKR, 2 5 AS, IRF 1, ICE and IL 18  [ **IL18** ] .  Ref: 12794720 J Med Virol< MedlineTA>J Med Virol, 2003
- T bet deficient DCs were significantly impaired in their capacity to secrete IFN gamma  [ **IFNG** ]  after both stimulation with IL 12 alone or in combination with IL 18  [ **IL18** ] .  Ref: 12802010 Proc Natl Acad Sci U S A< MedlineTA>Proc Natl Acad Sci U S A, 2003
- Responsiveness of CD4 T cells to the IFN gamma  [ **IFNG** ]  inducing cytokines IL 12 and IL 18  [ **IL18** ]  is generally thought to be acquired only after stimulation via the TCR.  Ref: 12811838 Eur J Immunol, 2003
- Mice deficient in IFN gamma  [ **IFNG** ]  are not resistant to lethal endotoxemia but are resistant when treated with neutralizing antibodies to IL 18  [ **IL18** ]  and challenged with a lethal injection of some endotoxins.  Ref: 12792854 J Infect Dis, 2003
- Recent reports demonstrated that P. acnes treatment promotes IL 12 and IL 18  [ **IL18** ]  synthesis in mice inducing IFN gamma  [ **IFNG** ]  release, enhancement of IgG2a switch and inhibition of Th2 cell expansion.  Ref: 12880687 Immunol Lett, 2003
- Sepsis is initiated and perpetuated by the overzealous systemic production of proinflammatory cytokines such as tumor necrosis factor TNF alpha, interferon IFN gamma  [ **IFNG** ] , interleukin IL 12, and IL 18  [ **IL18** ]  sometimes resulting in excessive tissue injury and death.  Ref: 12874588 Surgery, 2003
- Thus, deformed cortical bone and a decreased turnover rate of lumbar trabecular bone are characteristic of IL 18  [ **IL18** ]  TG mice, and these features might be associated with the increased expression of IFN gamma  [ **IFNG** ]  and IL 18  [ **IL18** ]  in the bone marrow.  Ref: 12817749 J Bone Miner Res, 2003
- Moreover, MHC class II DR expressing PMNs were also proved to express a high affinity receptor for immunoglobulin E IgE FcepsilonRI and to produce tumour necrosis factor alpha, interferon gamma  [ **IFNG** ]  and interleukin 18  [ **IL18** ]  following a challenge with an anti MHC class II DR monoclonal Ab MoAb or anti DR antiserum.  Ref: 12791081 Transfus Med, 2003
- Because IFN gamma  [ **IFNG** ]  plays a key pathogenic role in the development of murine immunoinflammatory diabetes induced by multiple low doses of streptozotocin STZ we investigated the effect of negating the actions of endogenous IL 18  [ **IL18** ]  in this model by administering recombinant IL 18  [ **IL18** ]  binding protein Fc IL 18  [ **IL18** ]  bp Fc.  Ref: 12884303 Eur J ImmunolEur J Immunol, 2003
- IL 12, IL 18  [ **IL18** ] , and IFN gamma  [ **IFNG** ]  levels in supernatants of the liver MNCs stimulated with LPS in CLP mice were significantly higher than those in sham mice 7 days after the procedure.  Ref: 12874588 Surgery, 2003
- We examined the effect of PGE2 on the expression of ICAM 1 and B7 in the human mixed leukocyte reaction MLR in the presence or absence of IL 18  [ **IL18** ] .METHODS We measured the expression of ICAM 1, B7.1, and B7.2 on human monocytes by flow cytometry and determined the associated production of interferon gamma  [ **IFNG** ]  and IL 12 by enzyme linked immunosorbent assay.  Ref: 12829919 Transplantation, 2003
- Deformed cortical bone and decreased turnover rate of lumbar trabecular bone are consistent with increased expression of IFN gamma  [ **IFNG** ]  and IL 18  [ **IL18** ]  in the bone marrow.  Ref: 12817749 J Bone Miner Res, 2003
- This study shows that inactivated parapoxvirus ovis Orf virus, strain D1701 PPVO, induces an autoregulatory cytokine response that involves the upregulation of IL 12, IL 18  [ **IL18** ] , IFN gamma  [ **IFNG** ]  and other T helper 1 type cytokines and their subsequent downregulation, which is accompanied by induction of IL 4.  Ref: 12810878 J Gen Virol< MedlineTA>J Gen Virol, 2003
- In chronic hepatitis C, but not in chronic hepatitis B nor in liver disorders unrelated to viral infections, IP 10 expression was strongly correlated with the amount of transcripts for IFN gamma  [ **IFNG** ]  and to the amount of transcripts for the constitutively expressed macrophage derived cytokine IL 18  [ **IL18** ] .  Ref: 12794718 J Med Virol, 2003
- Although IL 12 is also an IFN gamma  [ **IFNG** ]  inducing factor, the activity of IL 18  [ **IL18** ]  but not IL 12 in models of sepsis and death is dependent on the intracellular cysteine protease IL 1beta converting enzyme caspase 1.  Ref: 12792854 J Infect Dis, 2003
- Furthermore, serum IL 12 and IL 18  [ **IL18** ]  levels and liver MNCs IL 12, IL 18  [ **IL18** ] , and IFN gamma  [ **IFNG** ]  production were significantly increased in CLP mice compared with sham mice after LPS challenge.  Ref: 12874588 Surgery, 2003
- In this study, the authors observed that combined use of IL 18  [ **IL18** ]  and low dose IL 2 synergistically promoted in vitro proliferation of natural killer cells with up regulation of IL 2 receptor alpha and also synergistically stimulated cytolytic activity and interferon gamma  [ **IFNG** ]  production by these cells.  Ref: 12806277 J Immunother< MedlineTA>J Immunother, 2003
- Hepatic inflammatory activity was found to be associated more closely with IFN gamma  [ **IFNG** ]  than with IP 10 or IL 18  [ **IL18** ]  mRNA expression.  Ref: 12794718 J Med Virol, 2003
- ... IFN gamma  [ **IFNG** ] , IFNAR 1, STAT1alpha IRF 1, and IL 18  [ **IL18** ]  mRNA were upregulated.  Ref: 12794720 J Med Virol< MedlineTA>J Med Virol, 2003
- Whereas IL 18  [ **IL18** ]  significantly induced the expression of ICAM 1, B7.1, and B7.2 on monocytes in MLR and the production of interferon gamma  [ **IFNG** ]  and IL 12, PGE2 inhibited these IL 18  [ **IL18** ]  initiated enhancements.  Ref: 12829919 Transplantation, 2003
- uNK cells proliferate, produce cytokines interferon gamma  [ **IFNG** ]  IFN gamma  [ **IFNG** ]  and interleukin 18  [ **IL18** ]  IL 18  [ **IL18** ]  and IL 27, and terminally differentiate into granulated lymphocytes.  Ref: 12887272 Reproduction, 2003
- Changes in the local expression of IL 1beta, IL 2, IL 4, IL 6, IL 8, IL 10, IL 12, IL 15, IL 18  [ **IL18** ] , TNF alpha, IFN gamma  [ **IFNG** ]  and TGF beta 2 genes in the uterine adnexa and endometrium were studied in women with pyoinflammatory adnexal diseases.  Ref: 12802428 Bull Exp Biol Med< MedlineTA>Bull Exp Biol Med, 2003
